# Summary of the best evidence for early mobilization following laparoscopic radical resection for colorectal cancer

**DOI:** 10.3389/fmed.2026.1967502

**Published:** 2026-10-21

**Authors:** Rong She, Xiu Jin, Ziqin Shi, Huaping Wei, Xiaoyue Wei, Zeya Wu

**Affiliations:** 1 School of Nursing, Gansu University of Chinese Medicine, Lanzhou, Gansu, China; 2 Department of Cardiology, The First Hospital of Lanzhou University, Lanzhou, Gansu, China; 3 Outpatient Department, The First Hospital of Lanzhou University, Lanzhou, Gansu, China, China; 4 School of Nursing, Lanzhou University, Lanzhou, Gansu, China

**Keywords:** best evidence, colorectal cancer, early mobilization, evidence-based medicine, radical resection for colorectal cancer

## Abstract

**Purpose:**

To synthesize the best available evidence on early mobilization following radical surgery for colorectal cancer, both domestically and internationally, with the aim of providing evidence-based guidance for clinical healthcare providers.

**Methods:**

We conducted a literature search using the “6S” evidence model to identify studies on early mobilization following colorectal cancer surgery. Given the limited number of studies exclusively focusing on laparoscopic approaches, we included evidence from broader elective colorectal surgery populations to provide comprehensive guidance. We searched relevant domestic and international websites, evidence-based databases, association websites, and primary literature databases via a computerized system. The search period spanned from the inception of each database to December 19, 2025. Two researchers independently assessed the quality of the literature using the Joanna Briggs Institute (JBI) quality assessment tool, and conducted evidence extraction and synthesis for the included studies.

**Results:**

A total of 19 studies were ultimately included, comprising 4 guidelines, 6 expert consensus documents, 3 systematic reviews, 1 evidence summary, and 5 randomized controlled trials. A total of 23 pieces of evidence were identified across five areas: core principles, preoperative preparation, key elements of activity management, postoperative activity protocols, and activity support.

**Conclusion:**

This study synthesizes the best available evidence regarding early mobilization following laparoscopic radical resection for colorectal cancer. It recommends that nursing staff tailor their approach to individual patients based on clinical circumstances and patient preferences to promote postoperative recovery and enhance the quality of clinical care.

**Systematic Review Registration:**

http://ebn.nursing.fudan.edu.cn/myRegisterList, identifier ES20259534.

## 1 Introduction

Globally, Colorectal cancer is the third most common cancer and the second leading cause of cancer-related deaths. In 2022, it was estimated that there were approximately 1.92 million new cases and about 0.9 million deaths from CRC worldwide (1). Laparoscopic radical resection has become the predominant minimally invasive surgical approach for colorectal cancer. It offers distinct advantages, including excellent intraoperative visualization, reduced surgical trauma, diminished postoperative pain, and a lower incidence of complications, and it has shown to significantly improve short-term postoperative outcomes in patients (2). Robotic-assisted systems may be more advantageous than traditional laparoscopy in complex dissections, but a 2023 randomized controlled trial found that its advantages in routine colorectal cancer surgery must be balanced against much higher costs, with comparable clinical and oncological outcomes (3).

Within the multimodal intervention strategy of enhanced recovery after surgery concept, early postoperative mobilization has been proven to be a critical component in promoting rapid patient recovery and improving clinical outcomes (4). It effectively promotes the recovery of gastrointestinal function, enhances muscle strength, improves cardiopulmonary function, and reduces the risks of venous thromboembolism and pulmonary infections, thereby playing a crucial role in shortening hospital stays and improving patients' quality of life (5, 6).

Kehlet et al. (7) published a consensus in 2005 that systematically established the core ERAS intervention framework for colorectal surgery for the first time. However, a gap remains between theory and practice concerning specific implementation strategies for early postoperative mobilization, including the optimal timing for initiation, criteria for escalating activity intensity, safety protocols, and multidisciplinary collaboration. There is an urgent need to develop systematic, standardized, and actionable consensus guidelines based on the best available evidence.

Therefore, this study systematically integrates high-quality domestic and international guidelines, expert consensus documents, systematic reviews, and randomized controlled trials to develop a standardized protocol for early mobilization following laparoscopic radical resection for colorectal cancer. It aims to provide evidence-based support for establishing scientific, safe, and efficient perioperative nursing practices in clinical settings.

### 1.1 Main findings

Compared with previous evidence summaries, our first key change is a significant shift in analgesic strategies for laparoscopic patients.

The second key change is to shorten the postoperative activity goals.

The third key change is the establishment of clear stop criteria.

The fourth key change emphasizes the importance of optimizing comprehensive support measures.

### 1.2 Patient and public contribution

No patients, service users, caregivers, or members of the public were involved in the design, conduct, analysis, interpretation, or manuscript preparation of this study.

## 2 Materials and methods

### 2.1 Problem establishment

Within the framework of the Problem Identification Tool (PIPOST) (8), developed by the JBI Evidence Center, the following evidence-based question is defined. The target population (P) consists of patients undergoing laparoscopic radical resection for colorectal cancer. The intervention (I) includes various activities related to early mobilization. The comparator (C) is conventional postoperative care or delayed mobilization, where applicable. The professionals (P) refer to specialists working in oncologic or colorectal surgery, including healthcare providers and rehabilitation specialists. Outcome measures (O) include gastrointestinal function, length of hospital stay, complication rates, activities of daily living (ADL), and physical function. The setting (S) is colorectal surgery. The type of evidence (T) encompasses guidelines, expert consensus statements, systematic reviews, evidence summaries, and randomized controlled trials.

### 2.2 Search strategy

Following the “6S” evidence hierarchy (Systems, Summaries, Synopses of Syntheses, Syntheses, Synopses of Studies, and Studies), the search prioritized higher level evidence resources such as evidence summaries and guidelines, and progressively extended to lower-level evidence including systematic reviews and original studies. Computer searches were conducted on the websites and databases of the National Institute for Health and Care Excellence (NICE), JBI EBP Database, Guideline International Network (GIN), Scottish Intercollegiate Guidelines Network (SIGN), National Comprehensive Cancer Network (NCCN), UpToDate, Yi Maitong Guidelines Network, CNKI, Wanfang Database, VIP Database, SinoMed, PubMed, Embase, Web of Science, Elsevier, and the Cochrane Library. The searches employed a combination of subject headings and free-text terms. Keywords included “colorectal cancer,” “colorectal tumor,” “colorectal neoplasm,” “colorectal carcinoma,” “laparoscopic radical resection for colorectal cancer,” “early mobilization,” “early activity,” “early physical therapy,” “early physiotherapy,” “early rehabilitation,” “early exercise,” “early training,” “accelerated ambulation,” “enhanced recovery after surgery,” “enhanced postsurgical recovery,” and “minimally invasive.” The search period spanned from the inception of each database to December 19, 2025. No language restrictions were applied; both Chinese and English language literature retrieved. Retrieved records were imported into reference management software, and duplicates were removed. Search terms were adapted according to each database' s syntax and indexing rules. The PubMed search strategy is shown in Table 1 as an example, and the full search strategies for each database are provided in Supplementary Table S1.

**Table 1:** Pubmed search strategy.

Search ID	Search Strategy
#1	“Colorectal Neoplasms”[MeSH Terms]
#2	“colorectal neoplasm*”[Title/Abstract] OR “colorectal tumor*”[Title/Abstract] OR “colorectal cancer*”[Title/Abstract] OR “colorectal carcinoma*”[Title/Abstract]
#3	“Early Ambulation”[MeSH Terms]
#4	“early ambulation”[Title/Abstract] OR “accelerated ambulation”[Title/Abstract] OR “early mobilization”[Title/Abstract] OR “early mobilit*”[Title/Abstract]
#5	“laparoscop*”[Title/Abstract] OR “minimally invasive”[Title/Abstract]
#6	“Enhanced Recovery After Surgery”[MeSH Terms] OR “enhanced postsurgical recovery”[Title/Abstract] OR “Enhanced Recovery After Surgery”[Title/Abstract]
#7	#1 OR #2
#8	#3 OR #4 OR #6
#9	#7 AND #8 AND #5

### 2.3 Inclusion and exclusion criteria

Inclusion criteria: (1) Study subjects were colorectal cancer patients who underwent laparoscopic radical resection of colorectal cancer. (2) Include guidelines, expert consensus statements, systematic reviews, evidence summaries, and randomized controlled trials published from the inception of each database to December 19, 2025 that address early mobilization following colorectal radical surgery; (3) Research content encompassed pre-activity assessments, contraindications for activity suspension, and specific protocols for early mobilization.

Exclusion criteria: (1) Studies with incomplete data or unavailable full texts; (2) Studies with low-quality evaluations.

### 2.4 Evaluation of the quality of the literature

Two independent researchers conducted a quality assessment of the included studies. The Appraisal of Guidelines for Research and Evaluation, Version II (AGREE II) (9) tool was used to assess guideline quality. The methodological quality of the expert consensus and systematic reviews was assessed using the evaluation criteria of the Australian JBI Center for Evidence-Based Healthcare, which included 6 criteria for the expert consensus (10) and 11 criteria for the systematic reviews (11). Quality assessment was conducted using the Critical Appraisal for Summaries of Evidence (CASE) tool (12), and the quality of randomized controlled trials was assessed using the corresponding evaluation criteria from the Center for Evidence-Based Healthcare (13).

### 2.5 Evidence extraction, summary, grading, and recommendation

Researchers extracted data using a self-designed evidence extraction form collecting information such as the first author, publication year, literature topic, source of literature, content of recommendations, level of evidence, and strength of recommendations. Data extraction followed these principles: 1) For evidence with similar wording, select the version with concise phrasing that aligns with professional terminology; 2) For complementary evidence, integrate based on logical relationships; 3) For conflicting evidence, prioritize inclusion based on recency and quality; 4) For independently stated evidence, retain the original phrasing. After synthesizing the evidence, the JBI Evidence Grading and Recommendation Level System (14) was used to determine the grade of the included evidence. Recommendations were categorized as Grade A (strong recommendation) or Grade B (weak recommendation). When disagreements arose among researchers, group discussions were held until consensus was reached. Recommendation strength was then assigned by integrating the evidence level with the JBI FAME framework (feasibility, appropriateness, meaningfulness, and effectiveness), clinical applicability, and safety considerations; therefore, the recommendation grade (A or B) does not directly correspond to the evidence level. To minimize the risk of double-counting, evidence from secondary sources (guidelines, systematic reviews, and the existing evidence summary) was treated as integrated summaries of the underlying primary studies, whereas individual randomized controlled trials were used mainly to supplement implementation-specific details such as activity protocols and safety thresholds; primary studies known to underpin an included systematic review were not counted as independent evidence.

### 2.6 Trial registration

Evidence-based Medicine Center of Fudan University (registration number: ES20259534).

## 3 Results

### 3.1 Search results

An initial literature search yielded 2,130 articles; after removing duplicates, 1,885 remained. After reviewing the titles, abstracts, and full texts, 19 articles were ultimately included in the analysis, comprising 4 guidelines, 3 systematic reviews, 6 expert consensus statements, 1 evidence summary, and 5 randomized controlled trials. The detailed literature screening process is shown in Figure 1, and the general characteristics of the included articles are presented in Table 1. The reporting of this review followed the PRISMA 2020 statement where applicable for evidence summaries.

**Figure 1:**
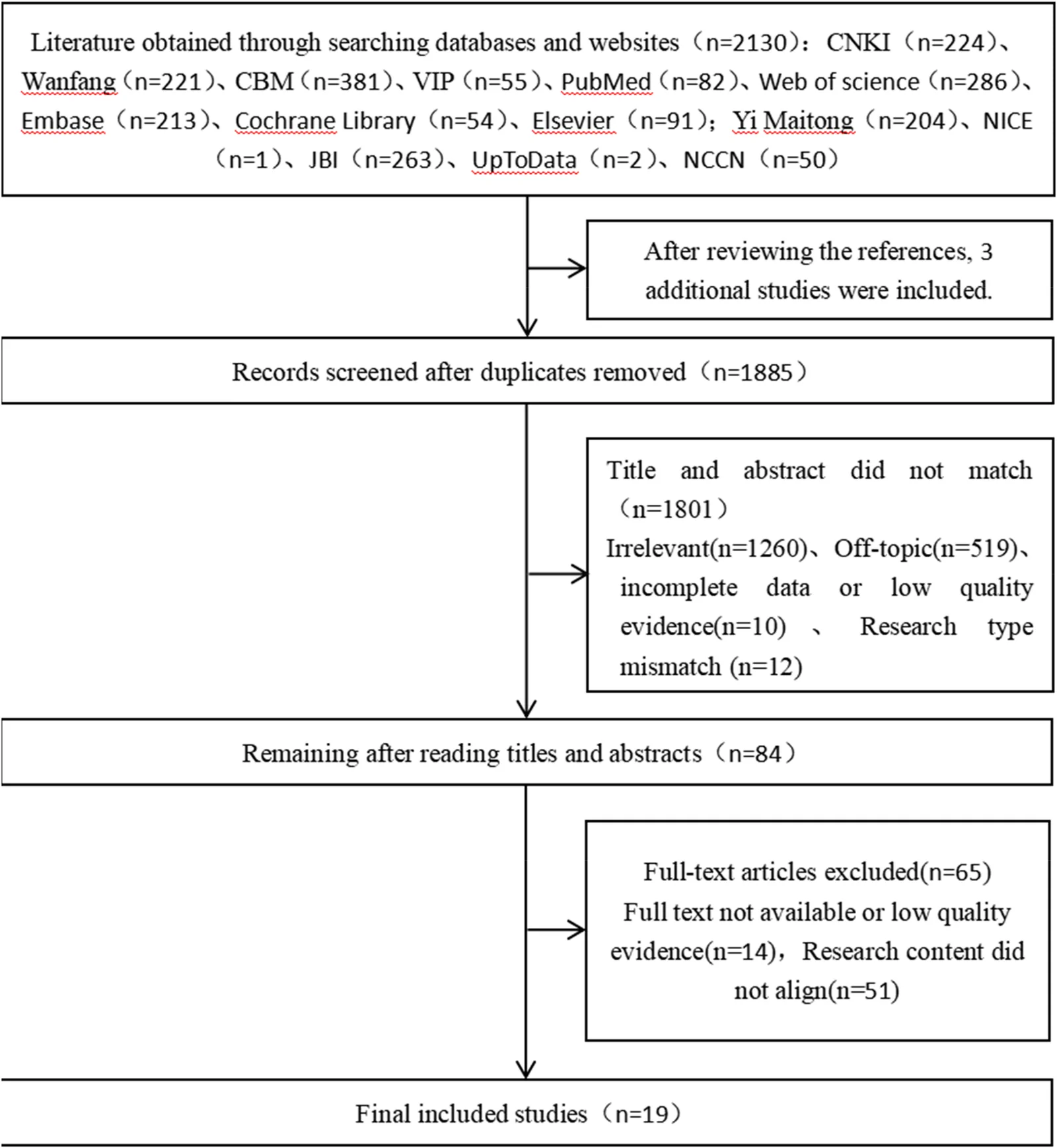
Flow diagram of literature screening.

### 3.2 Basic characteristics of included studies

The basic characteristics of all 19 included studies, including publication year, study type, title and literature source, are summarized below (see Table 2).

**Table 2:** General characteristics of the included literature (*n* = 19).

Included Literature	Year of publication	Type of literature	Title of literature	Literature source
Gustafsson UO et al. (15)	2025	Guideline	Guidelines for Perioperative Care in Elective Colorectal Surgery: Enhanced Recovery After Surgery (ERAS) Society Recommendations: 2025	PubMed
Irani JLet al. (16)	2023	Guideline	Clinical Practice Guidelines for Enhanced Recovery After Colon and Rectal Surgery From the American Society of Colon and Rectal Surgeons and the Society of American Gastrointestinal and Endoscopic Surgeons	Up to data
Chinese Society of Surgery, et al. (17)	2021	Guideline	Chinese Clinical Practice Guidelines for Enhanced Recovery After Surgery (2021 Edition)	Wan Fang
Chinese Society of Surgery, et al. (18)	2018	Guideline	Consensus on ERAS and guidelines for pathway management in China (2018)	Chinese Medical Journal
Liu et al. (19)	2020	Systematic review	Systematic Review and Meta-Analysis of Randomized Controlled Trials on the Efficacy of Enhanced Recovery After Surgery Management in Patients Undergoing Laparoscopic Colorectal Cancer Surgery	Wan Fang
Kannan V et al. (20)	2025	Systematic review	Impact of “enhanced recovery after surgery” (ERAS) protocols vs. traditional perioperative care on patient outcomes after colorectal surgery: a systematic review	PubMed
Li N et al. (21)	2023	Systematic review	Efficacy and safety of enhanced recovery after surgery pathway in minimally invasive colorectal cancer surgery: A systemic review and meta-analysis	Embase
Expert Panel for the Development of Cancer Rehabilitation Consensus (22)	2025	Expert consensus	Consensus Statement on Tumor Rehabilitation	Yi Maitong
Gu et al. (23)	2024	Expert consensus	Expert Consensus on Integrated Rehabilitation Management for Cancer (2024)	Yi Maitong
Chinese Society of Rehabilitation Medicine, Tumor Rehabilitation Professional Committee (24)	2023	Expert consensus	Expert Consensus on Functional Rehabilitation for Chinese Cancer Patients Focusing on Functional Impairment	Yi Maitong
Hedrick TL et al. (25)	2017	Expert consensus	American Society for Enhanced Recovery and Perioperative Quality Initiative Joint Consensus Statement on Postoperative Gastrointestinal Dysfunction Within an Enhanced Recovery Pathway for Elective Colorectal Surgery.	Yi Maitong
Zhejiang Colorectal Cancer Enhanced Recovery After Surgery (ERAS) Research Working Group (26)	2016	Expert consensus	Zhejiang Consensus on Comprehensive Enhanced Recovery After Surgery (ERAS) Protocol for Colorectal Cancer Based on Multicenter Clinical Research	Wan Fang
Laparoscopic & Endoscopic Surgery Group, Branch of Surgery, Chinese Medical Association, et al. (27)	2025	Expert consensus	Expert Consensus on Precision Surgical Diagnosis and Treatment of Colorectal Cancer (2025 Edition)	Yi Maitong
Luo et al. (28)	2022	Summary of Evidence	Summary of Best Evidence for Early Postoperative Mobilization in Patients Undergoing Colorectal Surgery	CNKI
Xiao et al. (29)	2021	Randomized controlled experiments	The Effectiveness of the Enhanced Recovery After Surgery Protocol in Perioperative Care for Laparoscopic Colorectal Cancer Patients	CNKI
Sun et al. (30)	2023	Randomized controlled experiments	Clinical Evaluation of Early Mobilization Protocol Following Single-Port Laparoscopic Surgery in Colorectal Cancer Patients	Wan Fang
LU Dongmei (31)	2021	Randomized controlled experiments	The Effect of Enhanced Recovery After Surgery Nursing on Recovery Outcomes and Early Ambulation in Patients Undergoing Laparoscopic Radical Resection for Colorectal Cancer	Wan Fang
Li M et al. (32)	2023	Randomized controlled experiments	Application and effect evaluation of precision nursing care of enhanced rehabilitation after surgery in the perioperative period of colorectal cancer	PubMed
Iqbal MT et al. (33)	2024	Randomized controlled experiments	Comparison of the outcomes of enhanced recovery after surgery (eras) vs conventional care in elective colorectal surgery	PubMed

### 3.3 Results of the quality evaluation of included literature

#### 3.3.1 Guideline quality evaluation results

Guidelines were classified as Grade A (strong recommendation) if six domains scored ≥60%, while a score of ≥30% in three or more domains qualified as Grade B (generally recommended) (34). Of the four guidelines included, one has a recommendation grade of “A” while the remaining three have a recommendation grade of “B”. The evaluation results are presented in Table 3.

**Table 3:** Quality evaluation results of guidelines.

Guideline	Percentage standardization by area (%)						≥60%	30%–60%	Recommended
	①	②	③	④	⑤	⑥			
Gustafsson UO et al. (15)	100%	83.3%	100%	100%	75%	100%	6	0	A
Irani JL et al. (16)	100%	83.3%	93.8%	94.4%	58.3%	100%	5	1	B
Chinese Society of Surgery, et al. (17)	100%	72.2%	48%	94.4%	37.5%	66.7%	4	2	B
Chinese Society of Surgery, et al. (18)	100%	55.6%	62.5%	88.9%	33.3%	16.7%	3	2	B

① Scope and purpose. ② Stakeholder involvement. ③ Rigor of development. ④ Clarity and presentation. ⑤Applicability. ⑥Editorial independence.

#### 3.3.2 Systematic evaluation or meta-analysis quality evaluation results

Three systematic reviews were included: one from the Wanfang Database, one from PubMed, and one from Embase. The evaluation results are presented in Table 4.

**Table 4:** Quality evaluation results of systematic reviews or meta-analyses.

Inclusion of literature	Evaluation Items										
	①	②	③	④	⑤	⑥	⑦	⑧	⑨	⑩	⑪
Liu Hui (19)	Yes	Yes	Yes	Yes	Yes	Yes	Yes	Yes	No	Yes	Yes
Kannan et al. (20)	Yes	Yes	Yes	Yes	Yes	unclear	Yes	Yes	No	Yes	Yes
Li et al. (21)	Yes	Yes	Yes	Yes	Yes	Yes	Yes	Yes	Yes	Yes	Yes

① Is the evidence-based question clear and well-defined? ② Are the inclusion criteria for the literature appropriate? ③ Is the search strategy appropriate? ④ Are the sources of the research articles appropriate? ⑤ Are the quality criteria for the literature appropriate? ⑥ Was the quality assessment of the literature conducted independently by two or more reviewers? ⑦ Were measures taken to minimize errors during data extraction? ⑧ Are the methods for synthesizing/combining studies accurate? ⑨ Was an assessment of potential publication bias conducted? ⑩ Were recommendations for policy and/or practice made based on the reported data? ⑪ Were appropriate suggestions made regarding specific directions for future research?.

#### 3.3.3 Quality evaluation results of the expert consensus

All items in the six expert consensus documents included in this study were rated as “Yes” The overall rating was “A” and all were included. All items in the six expert consensus documents included in this study were rated as “Yes” (Table 5). The overall rating was “A” and all were included.

**Table 5:** Quality evaluation results of expert consensus.

Expert consensus	Evaluation Items					
	a	b	c	d	e	f
Expert Panel for the Development of Cancer Rehabilitation Consensus (22)	Yes	Yes	Yes	Yes	Yes	Yes
Gu et al. (23)	Yes	Yes	Yes	Yes	Yes	Yes
Chinese Society of Rehabilitation Medicine, Tumor Rehabilitation Professional Committee (24)	Yes	Yes	Yes	Yes	Yes	Yes
Hedrick et al. (25)	Yes	Yes	Yes	Yes	Yes	Yes
Zhejiang Colorectal Cancer Enhanced Recovery After Surgery (ERAS) Research Working Group (26)	Yes	Yes	Yes	Yes	Yes	Yes
Laparoscopic & Endoscopic Surgery Group, Branch of Surgery, Chinese Medical Association (27)	Yes	Yes	Yes	Yes	Yes	Yes

a. Is the source of the viewpoint clearly cited? b. Does the viewpoint come from an influential expert in the field? c. Does the viewpoint prioritize the interests of the population under study? d. Are the conclusions based on the results of the analysis? Is the argument logically structured? e. Has other existing literature been referenced? f. Are there any inconsistencies between the proposed viewpoint and previous literature?.

#### 3.3.4 Results of the quality assessment of the evidence summary

This study included one evidence summary; items 3 and 8 were rated “No” while the remaining items were rated “Yes” as shown in Table 6.

**Table 6:** Quality evaluation results of evidence summary.

Evaluation Items	**①**	**②**	**③**	**④**	**⑤**	**⑥**	**⑦**	**⑧**	**⑨**	**⑩**
Luo et al. (28)	Yes	Yes	No	Yes	Yes	Yes	Yes	No	Yes	Yes

① Is the scope and application of the evidence summary specific? ② Is the author of the evidence summary transparent? ③ Are the reviewers/editors of the evidence summary transparent? ④ Are the search methods transparent and comprehensive? ⑤ Is the evidence grading system transparent and translatable? ⑥ Are the recommendations clear? ⑦ Can these recommendations be properly cited? ⑧ Are these recommendations up to date? ⑨ Is the evidence summary unbiased? ⑩ Can this evidence summary be applied to your patients?.

#### 3.3.5 Results of the quality assessment of randomized controlled trials

Of the five included RCTs, the study by Xiao et al. (29) was rated “Unclear” for items 2, 6, and 8; “No” for items 4 and 5; and “Yes” for all other items; Sun et al. (30) rated items 6 and 8 as “Unclear” items 4 and 5 as “No” and all other items as “Yes”; Lu Dongmei (31) rated items 2, 6, and 8 as “Unclear” items 4 and 5 as “No” and all other items as “Yes”; In the study by Li M et al. (32), item 6 was rated as “Unclear” items 4 and 5 as “No” and all other items as “Yes”; in the study by Iqbal MT et al. (33), items 2, 6, and 8 were rated as “Unclear” items 4 and 5 as “No” and all other items as “Yes” as shown in Table 7.

**Table 7:** Quality evaluation results of randomized controlled trials.

Evaluation Items	Xiao et al. (29)	Sun et al. (30)	Lu Dongmei (31)	Li M et al. (32)	Iqbal MT et al. (33)
①	Yes	Yes	Yes	Yes	Yes
②	Unclear	Yes	Unclear	Yes	Unclear
③	Yes	Yes	Yes	Yes	Yes
④	No	No	No	No	No
⑤	No	No	No	No	No
⑥	Unclear	Unclear	Unclear	Unclear	Unclear
⑦	Yes	Yes	Yes	Yes	Yes
⑧	Unclear	Unclear	Unclear	Yes	Unclear
⑨	Yes	Yes	Yes	Yes	Yes
⑩	Yes	Yes	Yes	Yes	Yes
⑪	Yes	Yes	Yes	Yes	Yes
⑫	Yes	Yes	Yes	Yes	Yes
⑬	Yes	Yes	Yes	Yes	Yes

①Was the allocation of study participants truly randomized? ②Was the allocation scheme concealed? ③Were the baseline characteristics of the experimental and control groups comparable? ④Were the study participants blinded? ⑤Were the intervention providers blinded? ⑥Were the outcome assessors blinded? ⑦Aside from the intervention being tested, were the other measures administered to participants in each group the same? ⑧Was follow-up complete? If not, were measures taken to address this? ⑨Were all enrolled participants included in the outcome analysis? ⑩Were outcome measures assessed using the same methods across all groups? ⑪Are the methods used to assess outcome measures reliable? ⑫Are the data analysis methods appropriate? ⑬Is the study design sound? Were there any deviations from standard RCT protocols during the conduct and analysis of the study?.

### 3.4 Summary and description of evidence

Evidence Summary on Early Mobilization Following Laparoscopic Radical Resection for Colorectal Cancer. The evidence summary for early mobilization after laparoscopic radical resection for colorectal cancer is shown in Table 8.

**Table 8:** Evidence summary on early mobilization following laparoscopic radical resection for colorectal cancer, including the category, evidence content, evidence level and recommendation level of each consensus statement.

Category	Evidence content	Evidence level	Recommendation level
Core Philosophy	1. Structured ERAS protocols should be promoted as standard of care, as they systematically improve outcomes for patients following laparoscopic colorectal cancer surgery (19–21).	1a	A
	2. The implementation of ERAS relies on the collaborative efforts of a multidisciplinary team—including surgeons, anesthesiologists, nurses, nutritionists, physical therapists, and psychologists—as well as standardized management (20).	1a	A
Preoperative Rehabilitation	3. Prior to surgery, patients and their families should receive health education that emphasizes the importance, specific components, and goals of early mobilization, with the aim of improving their understanding and adherence. Additionally, presenting success stories can help boost patients' confidence (17, 29).	1c	A
	4. Preoperative pre-rehabilitation focused on exercise therapy (cardiorespiratory exercise, aerobic exercise, and resistance training) can improve patients' functional reserve and exercise endurance, laying the foundation for postoperative rehabilitation; however, there is no standardized pre-rehabilitation protocol (15, 17, 22–24, 30, 32).	1c	B
	5. Physical function should be assessed prior to surgery using functional tests such as grip strength and the 6-minute walk test; these should be interpreted as measures of functional status rather than established predictors of surgical risk (17, 22, 24).	5b	A
Key Elements of Event Management	6. A personalized exercise prescription developed by a sports medicine or rehabilitation specialist based on the FITT principles (23).	5b	A
	7. A gradual, structured, and multi-stage approach should be followed, progressing from activities in bed to activities outside of bed (29–31).	1b	A
	8. Structured in-bed exercises should be initiated within 24 h after surgery, and patients should be encouraged to attempt standing or walking within 24 h (16, 26, 29, 30).	1b	A
	9. Starting on the first day after surgery, patients should be active for at least 3 h each day; by the time of discharge, patients should be able to move about independently or have regained their preoperative level of physical fitness (26).	5b	B
	10. High-risk patients whose surgical wounds have not yet healed must engage in physical activity under medical supervision (23).	5b	A
	11. Provisional indications for suspension: Pain score (VAS) > 4; oxygen saturation (SpO_2_) <90%; heart rate >100 bpm or < 40 bpm; abnormal blood pressure (systolic pressure <90 mmHg or diastolic pressure <50 mmHg); onset of dizziness, marked weakness, or orthostatic intolerance (30).	1c	B
	12. The effectiveness of the program should be evaluated using a combination of subjective reports and objective measurements. Indicators include time to first flatus, time to first bowel movement, time to mobilization, daily step count, distance walked, sleep duration, pain and fatigue scores, incidence of complications, and quality of life, among others (30).	1c	B
Postoperative Activity Plan	13. Prior to the procedure, assess the patients' level of consciousness, vital signs, pain level, muscle strength, and the stability of the incision, among other factors (30).	1c	A
	14. Chew gum 3–4 times a day for 10–15 min each time after surgery (16, 29).	1c	A
	15. Bed rest activities should begin within 24 h of regaining consciousness from anesthesia. These include deep breathing, elbow flexion exercises, ankle pumping exercises, knee flexion and hip elevation, and turning over in bed (29, 32).	1b	A
	16. During the transition phase, patients should gradually progress from bed rest to sitting up in bed, standing at the bedside, assisted walking, and finally independent walking, while gradually increasing the duration of each activity (29, 31).	1b	A
	17. Out-of-Bed Phase: Walk slowly with assistance, gradually increasing walking distance and duration (17, 29–32).	1b	A
	18. Gradually increase your activity level, take precautions to prevent falls, and avoid resistance training around the indwelling catheter (17, 24).	5b	B
	19. Postoperatively, a multimodal analgesia regimen is recommended with component-specific evidence: (a)a baseline of acetaminophen combined with bilateral transvs. abdominis plane(TAP) blocks (15–17); (b)for colonic surgery, selective COX-2 inhibitors may be used in conjunction, whereas NSAIDs should be avoided for rectal surgery (15, 16); (c)intrathecal morphine may be administered as needed to optimize visceral analgesia (15, 25).	1b	A
Event Support	20. For patients without high-risk factors, the urinary catheter should be removed within 24 h after surgery; for patients without a risk of urinary retention, the urinary catheter should be removed within 48 h after surgery. Other drainage tubes should be evaluated as soon as possible and removed at the appropriate time to minimize restrictions on the patient's mobility (15–17, 32).	1c	A
	21. Drugs such as dexamethasone, ondansetron, granisetron, lermosetron, and aprepitant should be used to prevent postoperative nausea and vomiting (15).	5b	B
	22. Conducting nutritional screening before surgery, monitoring nutrition daily after surgery, and initiating early enteral nutrition within 24 h of surgery, combined with oral nutritional supplements, can promote the recovery of gastrointestinal function and shorten the length of hospital stay (15, 23, 29, 31, 32).	1b	A
	23. The volume of postoperative intravenous fluids should be limited to prevent excessive fluid load from affecting the patient' s willingness and ability to get out of bed (26).	5b	B

## 4 Discussion

### 4.1 Early mobilization is a core component of the concept of enhanced recovery after surgery

Items 1–2 clarify the core principles of early postoperative mobilization following laparoscopic colorectal cancer surgery. Early postoperative mobilization is not a single intervention but rather a structured, progressive, and quantifiable implementation plan. Its core characteristic is a clearly defined timeframe, requiring that mobilization begin within 24 h postoperatively, consistent with the recommendations of the ERAS Society guidelines (35). Numerous ERAS guidelines, both domestic and international, list comprehensive pathways that incorporate early mobilization as the standard strategy for perioperative management of colorectal cancer (16, 17, 22–27, 35). Although the included randomized controlled trials exhibited some heterogeneity in terms of sample size and methodological details, their results generally indicated that structured early mobilization was associated with improved recovery of gastrointestinal function, earlier ambulation, and reduces the length of hospital stay in the short term, without an increased risk of adverse events (29–33). Nevertheless, the available evidence should be interpreted with caution. A randomized controlled trial of staff-directed facilitation of early mobilization within an enhanced recovery program for colorectal surgery found that, although in-hospital mobilization increased substantially, it did not improve walking capacity at 4 weeks or other measured clinical outcomes (36). Consistent with this, the 2025 ERAS Society guidelines characterize the evidence for specific mobilization interventions as limited, report no demonstrated improvement in functional capacity or complications for day-of-surgery mobilization, and rate the evidence for several outcomes of ≥3 h/day mobilization as very low (15). Throughout this manuscript, we therefore maintain the distinction between encouraging early mobilization to avoid the harms of prolonged immobility and the claim that a particular mobilization dose independently improves clinical outcomes.

### 4.2 Preoperative preparation is the foundation for implementing an early postoperative mobilization program

Items 3–5 focus on the preoperative preparation phase, the core of which lies in providing systematic health education to patients and their families. For patients with lower cognitive function, diverse educational strategies should be employed to deepen their awareness and understanding of early postoperative mobilization. However, in clinical practice, verbal instruction remains the primary method of health education; this approach can lead to poor retention of information by patients, which in turn affects their adherence to postoperative mobilization. Preoperative prehabilitation can enhance patients' functional reserve and exercise endurance, creating favorable conditions for postoperative rehabilitation; however, the existing evidence base remains relatively weak, lacking support from high-quality original research. Of note, preoperative assessments such as grip strength should be regarded as measures of functional status rather than established predictors of surgical risk. A prospective study of patients undergoing major hepatobiliary surgery found that impaired handgrip strength showed poor discriminatory ability for major postoperative morbidity, although it modestly predicted prolonged hospitalization (37).

### 4.3 Event management elements serve as a guide for the early stages of event implementation

Items 6–12 address key aspects of managing early postoperative mobilization, covering principles of mobility, mobility goals, indications for suspension, and outcome assessment. Specifically, Item 6–7 clarifies the principles for implementing mobility programs, emphasizing that the program should follow a progressive approach, gradually progressing along a standardized pathway from active bed exercises to sitting at the bedside, walking with assistance, and independent walking. This pathway has been validated and refined in multiple randomized controlled trials (RCTs) (29–31). Items 8–9 focus on activity goals for different stages of postoperative recovery and require that phased goals be established based on the patient's recovery progress during implementation; patients may only advance to the next phase of the program after meeting the requirements of the current stage. Items 10–12 address indications for suspending activities and the evaluation of outcomes. Activity safety is the cornerstone of implementing early mobilization programs; clear indications for suspension help clinicians identify abnormalities and promptly halt activities, thereby ensuring patient safety. At the same time, these criteria supplement the gaps in safety-related content found in previous evidence summaries (30, 32). However, because the proposed suspension thresholds derive largely from a single randomized controlled trial, they should be regarded as provisional, pragmatic criteria rather than established high-certainty thresholds, and their recommendation strength has been downgraded to weak (Grade B). Further validation through high-quality, multicenter studies is needed before they can be applied as universal safety standards.

### 4.4 The early mobilization plan serves as a guideline for patients' early postoperative mobilization

Items 13–19 summarize specific criteria for patient activity following laparoscopic radical resection for colorectal cancer; however, in clinical practice, these criteria should be individually tailored based on the patient's postoperative recovery. Before initiating activity, a comprehensive assessment of key indicators—including the patients' level of consciousness, vital signs, pain level, muscle strength, and incision healing—should be conducted. Only after ensuring that the patient meets the criteria for safe activity should step-by-step guidance be provided in accordance with predetermined activity goals. However, there remains a lack of sufficient consensus in the existing evidence regarding quantitative measures of activity intensity and activity protocols for patients with stomas.

### 4.5 Support measures for activities are a necessary prerequisite for implementing early-stage activities

Items 20–23 summarize supportive interventions for implementing early mobilization; these are essential safeguards for ensuring the safe implementation of early mobilization. Postoperative pain management is a fundamental prerequisite for early mobilization. Previous evidence has recommended thoracic epidural analgesia pumps or nonsteroidal anti-inflammatory drugs (NSAIDs) as the gold standard for pain management; however, the original studies on which this evidence is based primarily involved open or hybrid surgical approaches, and their pain management strategies may not fully align with the actual needs of patients undergoing laparoscopic surgery. Therefore, this study focuses on patients who have undergone laparoscopic colorectal radical resection and recommends a multimodal analgesic strategy centered on acetaminophen combined with bilateral transvs. abdominis plane (TAP)blocks, with selective COX-2 inhibitors added for colonic surgery and NSAIDs avoided for rectal surgery, and intrathecal morphine administered as needed to optimize visceral analgesia, thereby achieving effective pain control while preserving the colon-vs.-rectal distinction in the underlying evidence (16, 25). In addition, limiting intravenous fluid intake during and after surgery and encouraging early postoperative oral intake help maintain metabolic and internal homeostasis and conserve energy for physical activity (17, 26, 35). Removing the catheter as early as possible can reduce physical limitations at their source and help improve patients' mobility and willingness to move (27, 32).

The implementation of early postoperative mobilization is influenced by multiple factors. Age, health beliefs, the site and number of catheters, duration of anesthesia, severity of incision pain, and preoperative hemoglobin levels are all important factors affecting early postoperative mobilization (38). Other studies have shown that patients with spouses tend to have relatively higher adherence to postoperative activity guidelines (39). At the same time, a growing number of observational studies suggest a positive association between increased physical activity and improved health outcomes (40). However, the lack of healthcare personnel to assist patients with getting out of bed, as well as heavy clinical workloads, remain major obstacles to the effective implementation of activity-based rehabilitation programs (41).

Moreover, multidisciplinary quality-improvement programs have demonstrated the feasibility of achieving and sustaining high early mobilization rates in real-world settings. A multidisciplinary Clinical Practice Improvement Program (CPIP) for patients undergoing elective major hepatopancreatobiliary (HPB) surgery, using structured barrier analysis followed by Plan–Do–Study–Act cycles, increased the postoperative day (POD) 2 mobilization rate from 23% to 78.9%, sustained at 6 months, and reduced the median length of hospital stay from 8 to 6 days; wound pain was the most common reason for failure to mobilize on POD 2 (42). A subsequent prospective study confirmed the sustainability of this program during the COVID-19 pandemic, with the median mobilization rate maintained at 79% despite reduced staffing and resource availability, using explicit mobilization targets (sitting out of bed for >6 h on POD 1 and walking ≥30 m on POD 2) and reporting no falls or accidental drain dislodgements; pain and lethargy or giddiness were the most common reasons for failing to reach the target (43). Complementary evidence from colorectal surgery itself confirms the need for such structured support: in a single-center cohort of 1,170 patients managed within an established ERAS protocol, 58% did not mobilize as recommended (at least 6 h out of bed on POD1), with emergency surgery, advanced age, and intraoperative fluid overload identified as independent risk factors for delayed mobilization; delayed mobilization was in turn associated with more overall, major, and respiratory complications as well as a longer hospital stay (44). Although the two CPIP studies involved HPB rather than colorectal surgery and thus constitute indirect population evidence, together with the colorectal cohort they provide directly relevant implementation evidence concerning barriers, multidisciplinary support, adherence, and the sustainability of early mobilization programs, and their findings are consistent with the recommendation of this summary.

## 5 Conclusion

This study systematically summarizes the best evidence regarding early mobilization following laparoscopic radical resection for colorectal cancer, covering five aspects: core principles, preoperative preparation, key elements of mobilization management, postoperative mobilization protocols, and support for mobilization. A total of 23 evidence items were identified, providing evidence-based guidance for clinical healthcare professionals implementing early postoperative mobilization. Compared with previous evidence syntheses, this study focuses on the specific patient population undergoing laparoscopic radical resection for colorectal cancer, and its postoperative analgesia strategy has been adjusted accordingly to account for the characteristics of the laparoscopic approach, making it more targeted.

However, this study has certain limitations. First, the sample sizes of the included RCTs were limited, and the studies were primarily single-center, which to some extent limits the generalizability of the conclusions. Second, the existing evidence primarily focuses on short-term postoperative physiological indicators, with insufficient attention paid to patients' medium- and long-term quality of life, functional independence, and health economic benefits. Third, there is significant heterogeneity among studies regarding the specific intensity, frequency, and duration of activity programs. Fourth, because studies conducted exclusively in patients undergoing laparoscopic radical resection were limited, part of the included evidence originated from broader colorectal surgery and cancer rehabilitation populations; although this evidence was applied to the laparoscopic population with clinical judgment, its direct transferability warrants confirmation. Future large-sample, multicenter prospective studies or dose-response studies are needed to further clarify the optimal regimen for early postoperative mobilization. In terms of clinical translation, there remains a scarcity of high-quality original research on improving adherence to early mobilization protocols. In the future, wearable devices and mobile health technologies could be utilized to enable objective and subjective assessments of patients' activity levels, as well as continuous monitoring and intelligent feedback, thereby promoting the refinement and individualization of postoperative rehabilitation management.

## Data Availability

The original contributions presented in the study are included in the article/supplementary material, further inquiries can be directed to the corresponding authors.
