# Supplementary material for: Summary of the best evidence for early mobilization following laparoscopic radical resection for colorectal cancer
Source: Front Med (Lausanne). 2026 Oct 21;13:1967502. doi: 10.3389/fmed.2026.1967502 (PMC13730898; doi:10.3389/fmed.2026.1967502)
Supplement: Supplementary file 1 [file Table1.docx]

**Supplementary Table S1. Search strategies for all databases**

*Note: The PubMed search strategy is provided in Table 1 of the main text as an example. Full search strategies for each database/website are to be inserted in the "Search strategy" column below. Search period: from the inception of each database to December 19, 2025; no language restrictions were applied (both Chinese- and English-language literature were retrieved).*

| **No.** | **Database / Website** | **Search strategy** | **English translation** | **Search date** |
| --- | --- | --- | --- | --- |
| 1 | National Institute for Health and Care Excellence (NICE) | ("Colorectal Neoplasm*" OR "Colorectal Tumor*" OR "Colorectal Cancer*" OR "Colorectal Carcinoma*") AND ("Early Ambulation" OR "Accelerated Ambulation" OR "Early Mobilization" OR "Early Mobilit*") AND ("Enhanced Postsurgical Recovery" OR "Enhanced Recovery After Surgery" OR ERAS) AND ("laparoscop*" OR "minimally invasive") |  | Inception–19 Dec 2025 |
| 2 | JBI EBP Database | ("Colorectal Neoplasm" OR "Colorectal Tumor" OR "Colorectal Cancer" OR "Colorectal Carcinoma")  AND ("Early Ambulation" OR "Early Mobilization" OR "Early Ambulation" OR "early postoperative mobilization" OR "Enhanced Recovery After Surgery" OR "ERAS" OR "fast track" OR "enhanced recovery" OR "accelerated rehabilitation" OR "enhanced recovery of surgery program" )  AND（"guideline" OR "consensus" OR "systematic review"） |  | Inception–19 Dec 2025 |
| 3 | Guideline International Network (GIN) | "guideline" OR "consensus" OR "systematic review" |  | Inception–19 Dec 2025 |
| 4 | National Comprehensive Cancer Network (NCCN) | ("Colorectal Cancer" OR "Colon Cancer" OR "Rectal Cancer") AND ("Enhanced Recovery" OR ERAS OR "Early Ambulation" OR "Early Mobilization") |  | Inception–19 Dec 2025 |
| 5 | UpToDate | colorectal cancer AND enhanced recovery after surgery |  | Inception–19 Dec 2025 |
| 6 | Yi Maitong Guidelines Network | “加速康复外科 AND 腹腔镜结直肠癌 AND 早期活动” | “Enhanced Recovery After Surgery AND Laparoscopic colorectal cancer AND Early Mobilization ” | Inception–19 Dec 2025 |
| 7 | CNKI | （主题：结直肠癌 + 结直肠肿瘤 + 大肠肿瘤 + 结肠直肠癌）AND（主题：腹腔镜 + 微创） AND （主题：根治术 + 切除术） AND （主题：活动 + 下床活动 + 功能锻炼 + 早期活动 + 离床活动 + 加速移动 + 早日下床活动 + 加速康复外科 + ERAS） | (Subject: Colorectal cancer + Colorectal tumors + Large bowel tumors + Colorectal cancer) AND (Subject: Laparoscopy + Minimally invasive surgery) AND (Subject: Radical resection + Resection) AND (Subject: Activity + Ambulation + Functional exercise + Early mobilization + Out-of-bed activity + Accelerated mobility + Early ambulation + Enhanced Recovery After Surgery + ERAS) | Inception–19 Dec 2025 |
| 8 | Wanfang Database | 主题:(活动 OR 下床活动 OR 功能锻炼 OR 早期活动 OR 离床活动 OR 加速移动 OR 早日下床活动 OR 加速康复外科 OR ERAS) and 主题:(结直肠癌 OR 结直肠肿瘤 OR 大肠肿瘤 OR 结肠直肠癌) and 主题:(腹腔镜 OR 微创) and 主题:(根治术 OR 切除术) | Topic: (activity OR ambulation OR functional exercise OR early mobilization OR bed-exit activity OR accelerated mobility OR early ambulation OR Enhanced Recovery After Surgery OR ERAS) AND Topic: (colorectal cancer OR colorectal tumor OR colorectal neoplasm OR colorectal cancer) AND Topic: (laparoscopy OR minimally invasive surgery) AND Topic: (radical resection OR resection) | Inception–19 Dec 2025 |
| 9 | VIP Database | (篇关摘=结直肠癌 或者 篇关摘=结肠直肠癌 或者 篇关摘=结直肠肿瘤 或者 篇关摘=大肠肿瘤) 并且 (篇关摘=早期活动 或者 篇关摘=加速康复外科 或者 篇关摘=下床活动 或者 篇关摘=早期下床活动 或者 篇关摘=离床活动 或者 篇关摘=下地活动 或者 篇关摘=ERAS) 并且 (篇关摘=共识 或者 篇关摘=指南 或者 篇关摘=系统评价 或者 篇关摘=证据总结) | (colorectal cancer; or colorectal tumor; or colorectal neoplasm; or large bowel tumor) AND (early activity; or enhanced recovery after surgery; or ambulation; or early ambulation; or out-of-bed activity; or ambulation; or ERAS) AND (consensus; or guideline; or systematic review; or evidence summary) | Inception–19 Dec 2025 |
| 10 | SinoMed | (("根治术"[常用字段:智能] OR "切除术"[常用字段:智能]) AND ((("活动"[常用字段:智能] OR "下床活动"[常用字段:智能] OR "功能锻炼"[常用字段:智能] OR "早期活动"[常用字段:智能] OR "离床活动"[常用字段:智能] OR "加速移动"[常用字段:智能] OR "早日下床活动"[常用字段:智能]) OR ("运动(Movement)"[不加权:扩展])) OR (("加速康复外科"[常用字段:智能] OR "ERAS"[常用字段:智能]) OR ("术后加速康复"[不加权:扩展]))) AND (("微创"[常用字段:智能]) OR ("腹腔镜"[不加权:扩展])) AND (("结直肠癌"[常用字段:智能] OR "结肠直肠癌"[常用字段:智能] OR "大肠肿瘤"[常用字段:智能]) OR ("结直肠肿瘤"[不加权:扩展]))) AND ("循证文献"[文献类型] OR "临床试验"[文献类型] OR "随机对照试验"[文献类型] OR "Meta分析"[文献类型]) | "Radical resection" [Common field: Intelligent] OR "Resection" [Common field: Intelligent])AND((("Active" [Common field: Intelligent] OR "Out-of-bed activity" [Common field: Intelligent] OR "Functional exercise" [Common field: Intelligent] OR "Early mobilization" [Common field: Intelligent] OR "Out-of-bed activity" [Common field: Intelligent] OR "Accelerated mobility" [Common field: Intelligent] OR "Early out-of-bed activity" [Common field: Intelligent])OR("Movement" [Unweighted: Expanded])))OR(("Enhanced Recovery After Surgery" [Common field: Intelligent] OR "ERAS" [Common field: Intelligent])OR("Postoperative Enhanced Recovery" [Unweighted: Expanded]))))AND(("Minimally invasive" [Common field: Intelligent])OR("Laparoscopy" [Unweighted: Expanded])))AND(("Colorectal cancer" [Common field: Intelligent] OR "Colorectal carcinoma" [Common field: Intelligent] OR "Colorectal tumor" [Common field: Intelligent])OR("Colorectal neoplasm" [Unweighted: Expanded]))))AND("Evidence-based literature" [Document type] OR "Clinical trial" [Document type] OR "Randomized controlled trial" [Document type] OR "Meta-analysis" [Document type] | Inception–19 Dec 2025 |
| 11 | PubMed | ((Colorectal Neoplasms[Mesh]) OR (((((Colorectal Neoplasm [Title/Abstract])) OR (Colorectal Tumor [Title/Abstract])) OR (Colorectal Cancer [Title/Abstract])) OR (Colorectal Carcinoma[Title/Abstract]))) AND ((((((Early Ambulation[Title/Abstract])) OR ( Accelerated Ambulation [Title/Abstract])) OR (Early Mobilization[Title/Abstract])) OR (Early Ambulation[Mesh])) OR ((Enhanced Recovery After Surgery[Mesh]) OR (((Enhanced Recovery After Surgery [Title/Abstract])) OR (Enhanced Postsurgical Recovery[Title/Abstract])))) |  | Inception–19 Dec 2025 |
| 12 | Embase | #1=‘colorectal tumor’:ti,ab,kw OR ‘colorectal cancer’:ti,ab,kw OR ‘colorectal carcinoma’:ti,ab,kw  #2=‘colorectal tumor’/exp  #3=‘mobilization’:ti,ab,kw OR ‘early postoperative mobilization’:ti,ab,kw OR ‘accelerated ambulation’:ti,ab,kw  #4=‘mobilization’/exp  #5=‘enhanced postsurgical recovery’:ti,ab,kw OR ‘eras’:ti,ab,kw OR ‘fast track’:ti,ab,kw OR ‘enhanced recovery’:ti,ab,kw OR ‘accelerated rehabilitation’:ti,ab,kw OR ‘enhanced recovery of surgery program’:ti,ab,kw  #6=‘enhanced recovery after surgery’/exp  #7=#1 OR #2  #8=#3 OR #4  #9=#5 OR #6  #10=#8 OR #9  #11=#7 AND #10 |  | Inception–19 Dec 2025 |
| 13 | Web of Science | #1=(((TS=(Colorectal Neoplasms)) OR TS=(Colorectal Tumor)) OR TS=(Colorectal Cancer)) OR TS=(Colorectal Carcinoma)  #2=((((((((((TS=(Early Ambulation)) OR TS=(Early Mobilization)) OR TS=(early postoperative mobilization)) OR TS=(Accelerated Ambulation)) OR TS=(Enhanced Recovery After Surgery)) OR TS=(Enhanced Postsurgical Recovery)) OR TS=(ERAS)) OR TS=(fast track)) OR TS=(enhanced recovery)) OR TS=(accelerated rehabilitation)) OR TS=(enhanced recovery of surgery program)  #3=#1 AND #2 |  | Inception–19 Dec 2025 |
| 14 | Elsevier | ("Colorectal Neoplasm" OR "Colorectal Tumor" OR "Colorectal Cancer" OR "Colorectal Carcinoma") AND ("Early Ambulation" OR "Early Mobilization" OR "Early Ambulation" OR "Enhanced Recovery After Surgery" OR "ERAS" ) |  | Inception–19 Dec 2025 |
| 15 | Cochrane Library | #1=MeSH descriptor:[Colorectal Neoplasms] this term only  #2=Colorectal Tumor OR Colorectal Cancer OR Colorectal Carcinoma:ti,ab,kw  #3=MeSH descriptor:[Early Ambulation] this term only  #4=Early Mobilization OR early postoperative mobilization OR Accelerated Ambulation:ti,ab,kw  #5=MeSH descriptor:[Enhanced Recovery After Surgery] this term only  #6=Enhanced Postsurgical Recovery OR ERAS OR fast track OR enhanced recovery OR accelerated rehabilitation OR enhanced recovery of surgery program:ti,ab,kw  #7=#1 OR #2  #8=#3 OR #4  #9=#5 OR #6  #10=#8 OR #9  #11=#7 AND #10 |  | Inception–19 Dec 2025 |
